# Effects of deep neuromuscular blockade on the stress response during laparoscopic gastrectomy Randomized controlled trials

**DOI:** 10.1038/s41598-019-48919-2

**Published:** 2019-08-27

**Authors:** Bon-Wook Koo, Ah-Young Oh, Jung-Hee Ryu, Yea-Ji Lee, Ji-Won Han, Sun-Woo Nam, Do-Jung Park, Kwang-Suk Seo

**Affiliations:** 1 Department of Anesthesiology and Pain Medicine, Seoul National University Bundang Hospital, Seongnam, Korea; 2 Department of Anesthesiology and Pain Medicine, Seoul National University College of Medicine, Seoul, Korea; 3 Department of Surgery, Seoul National University College of Medicine, Seoul, Korea; 4 Department of Dental Anesthesiology, Seoul National University Dental Hospital, Seoul, Korea

**Keywords:** Randomized controlled trials, Characterization and analytical techniques

## Abstract

Maintaining deep neuromuscular block during surgery improves surgical space conditions. However, its effects on patient outcomes have not been well documented. We examined whether maintaining deep neuromuscular blockade during surgery could decrease the stress response compared to moderate neuromuscular blockade. Patients undergoing laparoscopic gastrectomy were randomly allocated to either the moderate (train-of-four counts of 1–2) or deep (post-tetanic counts of 1–2) neuromuscular blockade group. The primary outcome variable was the postoperative blood level of interleukin-6, and the secondary outcome variables were intraoperative or postoperative blood levels of tumor necrosis factor-α, interleukin-1β, interleukin-8, and C-reactive protein. A total of 96 patients were recruited and 88 (44 in each group) were included in the analyses. The levels of tumor necrosis factor-α and interleukin-1β measured at the end of surgery, interleukin-6 and interleukin-8 measured at 2 h postoperatively, and C-reactive protein measured at 48 h postoperatively were all significantly increased compared to the preoperative values, but there were no differences between the moderate and deep neuromuscular block groups. We found no differences in surgical stress response measured using determining levels of interleukin-6 and other mediators released between the moderate and deep neuromuscular blockade groups in patients undergoing laparoscopic gastrectomy.

## Introduction

The use of deep neuromuscular blockade (NMB) during surgery became practical with the introduction of sugammadex into clinical practice. The reversal of NMB with anticholinesterase depends on the depth of NMB at the time of reversal, and reversal of deep NMB is impractical. By contrast, use of sugammadex allows rapid reversal of even deep NMB^1^. The use of deep NMB during surgery has improved the surgical space conditions^2^. However, insufficient data are available regarding the beneficial effects of deep NMB itself on patient outcomes.

Surgery and related tissue injury induce changes in hemodynamic, metabolic, and immune responses, which are largely regulated by endogenous mediators called cytokines or endogenous hormonal responses. Postoperative levels of cytokines and acute-phase reactants are related to the extent of tissue injury and the occurrence of complications^3^. Previous reports have shown that the type of surgery and anesthetics used affect the degree of postoperative stress responses^4,5^. We hypothesized that improving the surgical conditions by maintaining intraoperative deep NMB may reduce related tissue damage and thereby reduce intraoperative and postoperative inflammatory mediators and acute-phase reactant release.

This study was performed to determine whether maintaining deep NMB during surgery would decrease the stress response compared to moderate NMB. The primary outcome variable was the level of interleukin (IL)-6 and the secondary outcome variables were the levels of tumor necrosis factor (TNF)-α, IL-1β, IL-8, and C-reactive protein (CRP).

## Results

**Figure 1:**
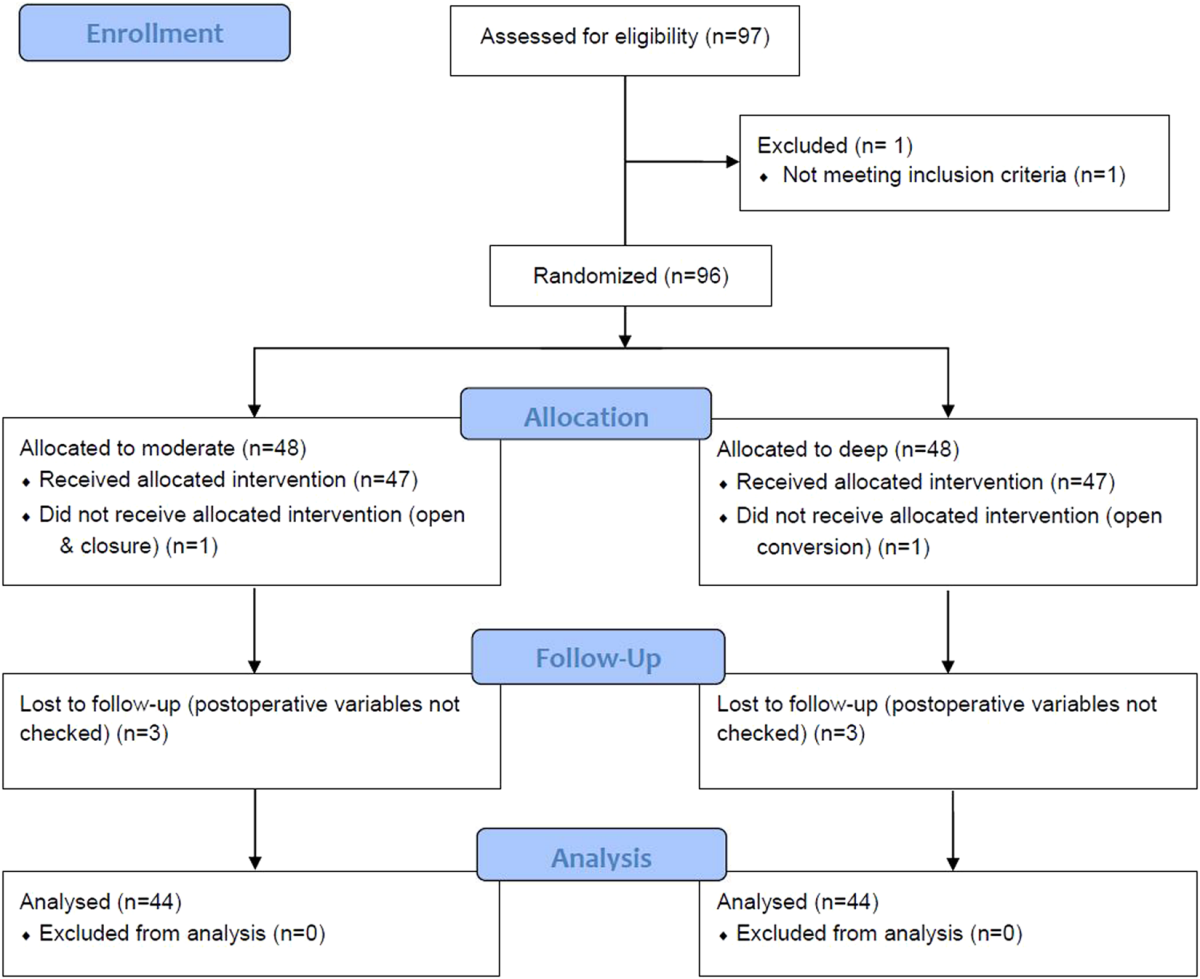
CONSORT diagram.

**Figure 2:**
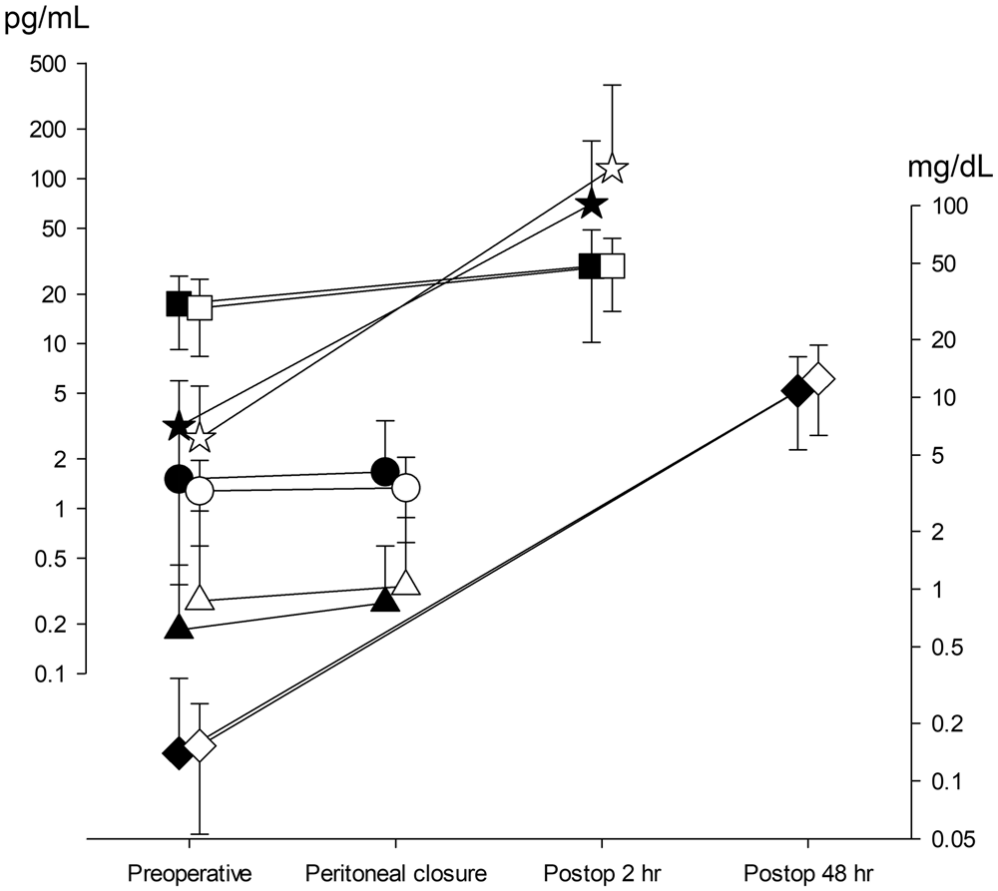
Surgical stress response estimated by TNF-α (● moderate, ○ deep) and IL-1β (▲ moderate, △ deep) at peritoneal closure, IL-6 (★ moderate, ☆ deep) and IL-8 (■ moderate, □ deep) at 2 h postoperatively, and CRP (♦ moderate, ◇ deep) at 48 h postoperatively. All of the levels were increased compared to the respective preoperative values (*P* < 0.05, Wilcoxon’s rank-sum test), but there were no differences between the moderate and deep groups (*P* > 0.05, Friedman’s test).

A total of 97 patients were assessed for eligibility. Of these, 1 did not meet the inclusion criteria and 96 patients were recruited. After allocation, the surgical plan was changed in two patients and six were lost to follow-up. Therefore, 88 patients were included in the analyses (Fig. 1). Patient characteristics, diagnosis, and types of operations were comparable between the two groups (Table 1). Restoration of spontaneous breathing (*P* = 0.006) and requests for additional NMB by the surgeon (*P* = 0.015) were more frequent in the moderate group compared to the deep group. Less rocuronium was used (*P* < 0.0001) but time to TOF 0.9 was significantly longer (*P* < 0.0001) in the moderate group compared to the deep group. The operation and anesthesia times were not different between groups (Table 2). The levels of TNF-α and IL-1β measured at the end of surgery, IL-6 and IL-8 measured at 2 h postoperatively, and CRP measured at 48 h postoperatively were all significantly increased compared to the respective preoperative values, but there were no differences between the groups (Fig. 2).

**Table 1:** Patient characteristics and diagnoses.

	Moderate (n = 44)	Deep (n = 44)	*p* value
Sex (M/F)	32/12	33/11	1.0
Age (years)	56 ± 11	59 ± 11	0.174
Weight (kg)	67.8 ± 10.7	65.8 ± 11.2	0.385
Height (cm)	166.4 ± 10.8	165.5 ± 8	0.635
BMI (kg/m^2^)	24.5 ± 3	23.9 ± 3.4	0.418
ASA (I/II)	32/12	24/20	0.12
**Diagnosis**			
Early gastric cancer	30	30	
Advanced gastric cancer	14	14	
**Operation**			
Totally laparoscopic distal gastrectomy	30	28	
Laparoscopic assisted distal gastrectomy	1	0	
Laparoscopic assisted proximal gastrectomy	7	10	
Laparoscopic assisted total gastrectomy	6	6	

Values represent the number or mean ± standard deviation. BMI, body mass index; ASA, American Society of Anesthesiologists.

**Table 2:** Intraoperative variables.

	Moderate (n = 44)	Deep (n = 44)	*P*
Spontaneous breathing (%)	11 (27.3)	2 (4.5)	0.006^¶*^
Requests for NMB (%)	32 (75)	23 (47.6)	0.015^¶^
Rocuronium (mg/kg)	2.0 ± 0.6	3.0 ± 1.1	<0.0001^†*^
Time to TOF 0.9 (min)	8.2 ± 5.0	3.8 ± 1.5	<0.0001^†*^
Operation time (min)	206.7 ± 49.7	210 ± 59.2	0.778^†^
Anaesthesia time (min)	250.3 ± 52.5	250.7 ± 58.3	0.974^†^

Values represent the number of patients (%) or mean ± standard deviation.

^¶^Chi-squared test, ^†^unpaired t-test, Asterisks indicate statistical significance (*p* < 0.05)

NMB, neuromuscular blockade; TOF, train-of-four ratio.

## Discussion

We found no differences in levels of IL-6 and other inflammatory cytokines, including TNF-α, IL-1β, and IL-8, and the acute-phase reactant CRP, perioperatively between the moderate and deep NMB groups of patients undergoing laparoscopic gastrectomy.

Surgery induces the stress response, consisting of sympathetic nervous system activation, endocrine response, immunological, and hematological changes, including cytokine production and acute-phase reaction^6^. Of these, we chose IL-6 as the primary outcome variable because it is the main cytokine responsible for inducing the systemic changes known as the acute-phase response^7^, the circulating levels of which are proportional to the extent of tissue injury during an operation^3,8^, is most consistently increased in the circulation of injured patients^9^, and is a predictor of morbidity after surgery^8^. Recently, the association between IL-6 and perioperative neurocognitive disorder has also been investigated^10,11^. The other cytokines measured in this study (i.e., TNF-α, IL-1β, and IL-8) and the acute-phase reactant CRP, are all interrelated. After acute injury, TNF-α and IL-1β are the earliest mediators released, followed by increases in the levels of IL-6 and IL-8, leading to an increase in CRP. We evaluated the levels of each mediator at different times, targeting the measurement to be at the peak level^3,8^.

Efforts to decrease the surgical stress response are ongoing. Lower concentrations of IL-6 and CRP were reported after laparoscopic surgery compared to open surgery^12^. Growth hormone level was lower in postoperative fast track care compared to standard care^8^. The neuroendocrine stress response in laparoscopic cholecystectomy was reduced to a greater extent in cases with spinal anesthesia compared to epidural anesthesia when combined with general anesthesia^5^. Several anesthetics such as isoflurane, sevoflurane, and propofol have also been studied with regard to neuroendocrine responses, but the results of these studies were inconsistent^13–15^. To the best of our knowledge, there have been no previous studies regarding the surgical stress response in relation to the depth of NMB.

The majority of studies that have investigated the effects of deep NMB have been performed during laparoscopic surgery. It seems obvious that deep NMB improves surgical space conditions during laparoscopic surgery^2,16^. Deep NMB reduces intraoperative patient movement and requests for additional NMB by the surgeon^16^, as also demonstrated in this study. That is, deep NMB improves both the satisfaction and convenience of surgeons and anesthetists. However, its effects on patient outcomes are less clear.

We postulated that the differences in surgical conditions due to deep NMB compared to moderate NMB may influence the extent of tissue injury and release of related mediators. We hypothesized that deep NMB would reduce tissue tension especially at the incisional sites and consequently reduce tissue damage. Our results showed improved surgical conditions by decreased incidental restoration of spontaneous breathing and decreased requests for NMB by the surgeons. However, we found no differences in the levels of cytokines or CRP between deep and moderate NMB groups. If we compare deep NMB with no NMB, instead of moderate NMB, the results might be different. Indeed, moderate NMB was recommended for surgical NMB until the introduction of sugammadex, which allowed clinical use of deep NMB. It seems necessary to find another way to show the beneficial effects of deep NMB on patient outcomes.

This study had several limitations. First, time to reach the peak concentrations of mediators after the insult and the extent of the insult may vary between patients. However, we measured the mediators at one predetermined time point; TNF-α and IL-1β at the end of peritoneal closure, IL-6 and IL-8 at 2 h postoperatively, and CRP at 48 h postoperatively. The measurement times were determined to target the peak concentrations reached based on previous reports, but these may vary between individual patients^3,8^. Second, the anesthetics used as well as surgery may have affected the release of mediators. Anesthetics have effects on the stress response, including the release of cytokines^13–15^. We controlled the dose of propofol and remifentanil according to the BIS value and the vital signs but did not measure the dose administered. Third, we attempted to control the level of NMB as deep or moderate in each group, but there would have been times outside of the middle of the range. This can be seen from the restoration of spontaneous breathing and requests for additional NMB by the surgeon during surgery in the deep group although the incidence was significantly lower than in the moderate group. TOF was measured continuously in the moderate group but PTC was measured at 6-minute intervals in the deep group taking into consideration the enhancing effect on subsequent muscle response. However, 3 min is sufficient to avoid the effect on subsequent muscle response after tetanic stimiulation^17,18^. Reducing the interval between monitoring of PTC to 3 min would help to maintain deep NMB.

In conclusion, deep NMB during laparoscopic gastrectomy improved surgical condition, as demonstrated by reduced incidences of intraoperative patient movement and requests for additional NMB by the surgeon. However, we found no evidence that deep NMB reduced the stress response by examining the levels of IL-6 and other mediators, including TNF-α, IL-1β, IL-8, and CRP.

## Methods

### Ethical approval and informed consent

This prospective, randomized, controlled study (B-1402/240-007) was approved on 13 March 2014 by the Ethical Committee of Seoul National University Bundang Hospital, Seongnam, South Korea (Chairperson prof. Lee Jae Ho) and registration at clinicaltrials.gov (NCT02100280_23/March/2014), and adhered to the Declaration of Helsinki. All subjects were completely informed consent.

### Anesthesia

Adult patients (≥18 years), with American Society of Anesthesiologists physical status I or II, scheduled for elective laparoscopic gastrectomy who provided written informed consent were included in the study. Patients with renal or hepatic dysfunction, history of neuromuscular disease, allergy to neuromuscular blocker, or family history of malignant hyperthermia were excluded. Patients were randomly assigned to moderate and deep NMB groups using computer-generated random numbers (Random Allocation Software, version 2.0). All of the primary and secondary outcome variables were checked by investigators blinded to the patient groups. When patients arrived at the reception area, premedication was performed with 0.03 mg/kg midazolam. Anesthesia was induced and maintained with intravenous (IV) propofol, remifentanil, and rocuronium. The dose of propofol was adjusted to maintain a bispectral index (BIS) (A-2000 BIS™ monitor; Aspect Medical Systems, Inc., Natick, MA, USA) value of 40–50, remifentanil to maintain blood pressure and heart rate within 20% of the preoperative value, and rocuronium to maintain train-of-four (TOF) 1–2 (moderate group) or post-tetanic count (PTC) 1–2 (deep group). Monitoring consisted of electrocardiography, non-invasive blood pressure, pulse oximetry, esophageal temperature, end tidal CO_2_, BIS.

### Neuromuscular monitoring

Acceleromyography (TOF-Watch-SX; MSD BV, Oss, The Netherlands) was applied to monitor the response of the adductor pollicis muscle. The patient’s position was supine with the arm opened using an arm board to prevent inadvertent touch by the surgeon. The forearm and fingers other than the thumb were fixed to prevent artefacts. Neuromuscular monitoring and management were performed according to the Good Clinical Research Practice guidelines (monitoring site: ulnar nerve/adductor pollicis muscle, stimulation pattern: TOF/2 Hz for 1.5 s repeated >12 s, Initial signal stabilization: calibration/7 step)^19^. After induction of anesthesia, continuous neuromuscular monitoring was started after calibration and stabilization of the signal as recommended in the Good Clinical Research Practice guidelines; 1. Apply a few stimulations (TOF, using 40–50 mA), 2. Apply a 50 Hz tetanic stimulation for 5 s, 3. Adjust twitch height to 100%, 4. Ensure supra-maximal stimulation, 5. Start the stimulation pattern and rate to be used in the study, 6. Recalibrate, if twitch height deviates from 100% by more than 5%, 7. Is stable baseline achieved within 2–5 min? Yes: administer NMBA No: check the equipment, repeat the set-up procedure and start again from ‘step 3’. After stabilization, 0.6 mg/kg rocuronium was administered IV for tracheal intubation. After the start of the operation, continuous IV infusion of rocuronium was begun at 20 mg/h and was adjusted to maintain TOF 1–2 (moderate block) or PTC 1–2 (deep block). TOF was measured every 15 s using TOF-Watch-SX in repetitive mode and PTC was measured every 6 min. The dose of rocuronium was adjusted in increments of 5 mg/h in all of the cases according to the measured neuromuscular response. At the end of surgery, NMB was reversed by IV neostigmine up to 50 μg/kg with glycopyrrolate in a 5:1 ratio in the moderate group and 2–4 mg/kg sugammadex IV in the deep group.

### Cytokine analysis

Blood samples were collected from the antecubital vein of the arm not used for IV infusion preoperatively for baseline measurements, at the end of peritoneal closure for TNF-α and IL-1β; at 2 h postoperatively for IL-6, IL-8; and at 48 h postoperatively for CRP. The blood samples were collected in serum separating tubes and were left at room temperature for more than 30 min followed by centrifugation at 3,000 rpm for 10 min. The separated serum samples were transferred to microtubes (1.0 mL each), and stored in a deep freezer (less than –20 °C). Cytokines were analysed using enzyme-linked immunosorbent assay kits (R&D Systems, Minneapolis, MN, USA) and CRP was determined using a chemistry analyser (Beckman Coulter, Brea, CA, USA).

### Outcomes

Intraoperative patient movement including restoration of spontaneous respiration, requests for additional NMB by the surgeon, the amount of rocuronium used, time from administration of reversal agent to TOF ratio 0.9, operation time, and anesthesia time were recorded.

### Statistical analysis

The primary outcome variable was the level of IL-6. To detect a difference in 10 pg/L in IL-6 level with α = 0.05 and β = 0.2, 43 patients per group were needed. This calculation was based on a between-subject standard deviation of change in 16.3 pg/mL for IL-6 level in a previous study. Considering the 10% dropout rate, a total of 48 patients per group were required. Data are expressed as the mean (SD) or median (IQR [range]) and *P* < 0.05 was considered statistically significant. Group comparisons were made using the unpaired *t*-test for continuous variables and the chi-square or Fisher’s exact test for dichotomous variables as appropriate. Differences between pre- and postoperative concentrations of TNF-α, IL-1β, IL-6, IL-8, and CRP were analyzed using the Wilcoxon rank-sum test and group comparisons were performed using Friedman’s test.

## Data Availability

The datasets generated during and/or analyzed during the current study are available from the corresponding author on reasonable request.
